# Supplementary material for: Glutaredoxins Rapidly Reduce Glutathione Hydroper‐ and Polysulfides
Source: Angew Chem Int Ed Engl. 2026 Sep 21;65(45):e1222129. doi: 10.1002/anie.1222129 (PMC13725587; doi:10.1002/anie.1222129)
Supplement: Supplementary file 1 [file ANIE-65-e1222129-s001.docx]

**Supporting Information**

**Glutaredoxins Rapidly Reduce Glutathione Hydroper- and Polysulfides**

**Philipp Reinert^1+^, Seiryo Ogata^2,3+^, Laura Leiskau^1^, Sernur Sena Yildiz^2^, Takaaki Akaike^3^, Uladzimir Barayeu^2,3^* and Marcel Deponte^1^***

^1^Department of Chemistry, Comparative Biochemistry, RPTU Kaiserslautern, D-67663 Kaiserslautern, Germany

^2^Max-Planck-Institute for Polymer Research, D-55128 Mainz, Germany

^3^Department of Redox Molecular Medicine, Tohoku University Graduate School of Medicine, Sendai 980-8575, Japan

^+^These authors contributed equally to this work.

*Corresponding author, orcid.org/0000-0001-8426-166X,

e-mail: [barayeuu@mpip-mainz.mpg.de](mailto:barayeuu@mpip-mainz.mpg.de)

*Corresponding author, orcid.org/0000-0003-2141-917X,

e-mail: [deponte@rptu.de](mailto:deponte@rptu.de)

KEYWORDS: Glutaredoxin • polysulfide • hydropersulfide • hydrogen sulfide • hydrogen polysulfides

**Materials and Methods**

**Materials**

The polysulfides GS_3_G, GS_4_G and CysS_3_Cys were synthesized as described previously.^[1]^ Diethylenetriaminepentaacetic acid (DTPA) was from Carl Roth, dithiothreitol (DTT), GSH, GSSG, 3-(dimethylcarbamoylimino)-1,1-dimethylurea (diamide), Na_2_S and ScGR were from Sigma-Aldrich, L-cystine was from Carbolution, NADPH was from Gerbu, *β*-(4-hydroxyphenyl)ethyl iodoacetamide (HPE-IAM) was from ChemCruz and formic acid was from Merck. Primers were purchased from Metabion. All restriction enzymes, Phusion HF DNA polymerase and T4 DNA li-gase were from New England Biolabs.

**Site-directed mutagenesis**

Plasmid pQE30/*PFGRX^C29S/C32S/C88S^* was generated by site-directed mutagenesis using *Pfu* po-ly-merase (Promega) with pQE30/*PFGRX^C32S/C88S^* as a template,^[2]^ and mutagenesis primers C29S_forward (5′-GTATTTGCAAAAACGGAAAGCCCATATAGTATTAAGGC-3′) and C29S_reverse (5′-GCCTTAATACTATATGGGCTTTCCGTTTTTGCAAATAC-3′). The gene encoding PfGrx^NC^ was generated by site-directed mutagenesis using Phusion HF DNA polymerase with pQE30/*PFGRX^C29S/C32S/C88S^* as a template and mutagenesis primers E28W_forward (5′-GCTGTATTTGCAAAAACGTGGAGCCCATATAGTATTAAGG-3′) and E28W_reverse (5′-CCTTAATACTATATGGGCTCCACGTTTTTGCAAATACAGC-3′). The methylated template DNA was digested with *Dpn*I and the PCR product was transformed into chemically competent *E. coli* XL1-Blue cells. Plasmid DNA was isolated by minipreparation and all constructs were verified by Sanger sequencing (Microsynth Seqlab). Models of oxidized dithiol tryptophan mutant PfGrx^E28W/C88S^ (PfGrx^DT^) and reduced monothiol tryptophan mutant PfGrx^E28W/C32S/C88S^ (PfGrx^DT^) were visualized following the introduction of point mutations using Swiss PDB viewer^[3]^ based on the structure of wild-type PfGrx (PDB entry 4MZB)^[4]^.

**Heterologous expression, protein purification and sample preparation**

Recombinant *N*-terminally MRGSH_6_GS-tagged PfGrx^DT^, PfGrx^MT^ and PfGrx^NC^ were produced in *E. coli* XL1-Blue cells and purified via affinity chromatography using nickel-nitrilotriacetic acid (Ni-NTA) agarose as described previously.^[5-6]^ Fully reduced proteins were obtained by incubating the eluates from the Ni-NTA agarose column with 5 mM DTT for 30 min on ice. Excess DTT and imidazole were removed on a PD-10 desalting column (Cytiva) using 3.5 mL ice-cold assay-buffer (100 mM Na_x_H_y_PO_4_, 0.1 mM DTPA, pH 7.4 at 25 °C) for elution. To obtain PfGrx^DT^(S_2_) or PfGrx^MT^(SSG), the reduced proteins were incubated with 5 mM diamide or 5 mM GSSG for 1 h on ice and again purified using Ni-NTA agarose and PD-10 columns as described above. The purity of the final eluates was confirmed by analytical SDS-PAGE (Suppl. Fig. S8) and protein concentrations were determined spectrophotometrically at 280 nm. Extinction coefficients of 15.60 mM^−1^cm^−1^ for PfGrx^DT^(S)_2_ and 15.47 mM^−1^cm^−1^ for all other PfGrx variants were calculated from the amino-acid sequence using the Expasy ProtParam tool (https://web.expasy.org/protparam). Concentrations for ScGR in ice cold assay buffer refer to the concentration of active sites and were determined based on an extinction coefficient at 462 nm of 12.9 mM^−1^cm^−1^.^[7]^

**Fluorescence spectra**

Fluorescence spectra of PfGrx^MT^ were recorded in a thermostatted SX-20 spectrofluorometer (Applied Photophysics) at 25°C using freshly purified enzyme. The tryptophan fluorescence was observed as the total emission at variable excitation wavelengths using a cut-off filter at 320 nm and a slit width of 2 mm. The spectra of reduced and glutathionylated PfGrx^MT^ were obtained 5 min after mixing 2 µM reduced PfGrx^MT^ in syringe 1 with either assay buffer or 120 µM GSSG in syringe 2.

**Stopped-flow fluorescence kinetic measurements**

Stopped-flow fluorescence kinetic measurements were performed at 25°C using freshly purified enzyme. The change of tryptophan fluorescence was observed for up to 300 s as the total emission at an excitation wavelength of 295 nm using a cut-off filter at 320 nm and a slit width of 2 mm. The reaction of reduced PfGrx with GSSG, GS_3_G, GS_4_G, cystine or CysS_3_Cys was investigated by mixing 0.5–1 µM enzyme in syringe 1 with variable concentrations of substrate in assay buffer in syringe 2. Likewise, the reaction of reduced PfGrx with *in situ*-generated GSSH or GS_3_H was measured by mixing 0.5–1 µM enzyme in syringe 1 with 0.12 µM ScGR, variable concentrations of GS_3_G or GS_4_G and 1 equivalent NADPH in syringe 2. The incubation-time for the solution in syringe 2 prior to mixing was ~1 min at 25°C to ensure the complete conversion of the polysulfides to hydroper/polysulfides by ScGR. To investigate the reaction of oxidized PfGrx with Na_2_S or DTT, 1–2 µM PfGrx^DT^(S_2_) or PfGrx^MT^(SSG) in syringe 1 was mixed with variable concentrations of Na_2_S or DTT in syringe 2. Traces of 2–3 consecutive measurements were averaged and fitted by single-, double- or triple-exponential regression using the Pro-Data SX software (Applied Photophysics) to obtain *k*_obs_ values. The *k*_obs_ values of up to three biolo-gical replicates from independent protein purifications were averaged and plotted against the substrate concentration in SigmaPlot 13.0 to determine second-order rate constants from the slopes of the linear fits.

**Stopped-flow absorbance kinetic measurements**

Stopped-flow absorbance kinetic measurements were performed at 25°C using ScGR. The consumption of NADPH was monitored at 340 nm for up to 1000 s using assay buffer as a blank, a slit width of 0.5 mm and an extinction coefficient of 6.22 mM^−1^cm^−1^. The reduction of GSSG, GS_3_G or GS_4_G by ScGR was investigated by mixing 0.1 mM substrate in syringe 1 with 1, 2 or 3 equivalents NADPH and 0.60 µM ScGR in syringe 2. Assays with assay buffer in syringe 1 served as negative controls. Traces of two consecutive measurements were averaged and plotted in SigmaPlot 13.0. Competition experiments between reduced ScGR and PfGrx^MT^ for the reduction of GS_3_G and GSSH were performed with 100 µM GS_3_G in syringe 1 and a mixture of 0.60 µM ScGR, 300 µM NADPH, 10 mM DTT and variable concentrations of reduced PfGrx^MT^ in syringe 2. Measurements without Grx and/or DTT served as controls. The consumption of NADPH (Δ[NADPH]) during phase 1 and 2 was calculated from the change of absorbance and plotted against the [Grx]/[GR] ratio. Curves were then fitted by a hyperbolic decay function f(x) = (*y*_0_ × *K*_1/2_) / (*K*_1/2_ + x) with *y*_0_ being the y-axis intercept and *K*_1/2_ being the [Grx]/[GR] ratio at half maximal amplitude (f(*K*_1/2_) = *y*_0_/2).

**Whole-protein mass spectrometry**

Mass spectrometry samples were obtained following incubation of 10 µM reduced PfGrx^MT^ or PfGrx^DT^ with 10–250 µM substrate for up to 15 min on ice as indicated. GSSH or GS_3_H were prepared *in situ* as described above. Reference spectra were recorded for re-purified and desalted 10 µM reduced or *S*-glutathionylated PfGrx^MT^ as described above. All samples were incubated with 0.5 or 2 mM HPE-IAM for 20 min at 37°C. Samples were then diluted 1:10 with 0.1% formic acid and stored at −80°C. Whole-protein electrospray mass spectrometry was conducted in positive-ion mode using a liquid chromatography system with a POROS 10R1 column and a Bruker maXis time-of-flight spectrometer as described previously.^[8]^

**Quantitative low-molecular-weight mass spectrometry**

Low-molecular-weight mass spectrometry samples for PfGrx were prepared as for whole-protein mass spectrometry. In case of ScGR, 0.30 µM enzyme was incubated with 10 µM GS_3_G or GS_4_G and 1 or 2 equivalents NADPH for up to 30 min at 25°C. Negative controls included samples without enzyme. All samples were incubated with 0.5 mM HPE-IAM for 20 min at 37 °C. Samples were then diluted 1:10 with 0.1% formic acid and stored at −80°C. For quantitative low-molecular-weight mass spectrometry, samples were further diluted 1:1 with 0.1% formic acid containing known amounts of isotope‐labeled internal standards. Analyses were performed on a LC-ESI-QTOF mass spectrometer (SYNAPT G2-Si, Waters) coupled to an ACQUITY UPLC system (Waters). Each sulfur metabolite was identified and quantified using targeted MS/MS mode. The synthesis of isotope-labelled internal standards, LC-ESI-MS conditions and targeted MS/MS parameters were applied as described previously.^[9-10]^

**Supplementary Figures**

**Figure S1**

**Supplementary Figure S1 | Control experiments for stopped-flow measurements.** **A**) Representative fluorescence spectra of reduced and glutathionylated PfGrx^MT^ recorded with the stopped-flow spectrofluorometer. **B**) Re-presentative stopped-flow kinetics for the absent reaction of cysteine-free negative control PfGrx^E28W/C29S/C32S/C88S^ (PfGrx^NC^) with the indicated oxidants at 25°C and pH 7.4. **C**) Rate constant for a second GSSG-dependent reaction phase of PfGrx^MT^ in Fig. 2A, which might reflect the reverse reaction.

**Figure S2**

**Supplementary Figure S2 | Reaction kinetics for the PfGrx^MT^-dependent reduction of cystine and CysS_3_Cys.** **A**) Representative stopped-flow kinetics for the reaction of reduced PfGrx^MT^ with variable concentrations of cystine (left) or CysS_3_Cys (right) at 25°C and pH 7.4. Control experiments with cysteine-free PfGrx^NC^ are shown in red. **B**) Secondary plot for the *k*_obs_ values from single exponential fits from panel A. The cystine-dependent rate constant was determined from the slope of the secondary plot. Representative traces in panel A were averaged from two technical replicates and *k*_obs_ values in panel B were from a single experiment (*n* = 1×2). **C**) Secondary plots for the *k*_obs_ values from single exponential fits from panel A. The CysS_3_Cys-dependent rate constant was determined from the slope of the secondary plots. Representative traces in panel A were averaged from two technical replicates and *k*_obs_ values in panel C were generated from three independent biological replicates (*n* = 3×2). **D**) Whole-protein mass spectrometry after incubation of CysS_3_Cys with reduced PfGrx^MT^ for 10 s on ice. The calculated mass for reduced PfGrx^MT^ is 13888 Da (following treatment with 2 mM HPE-IAM). Additional peaks may arise from oxidation of argininyl to glutamyl residues (−27 Da), methylation (+14 Da), potassium adducts (+38) or alkylation of amines by HPE-IAM (+178 Da). The peak at 13831, which is close to the calculated mass of cysteinylated PfGrx^MT^ of 13830 Da, was also detected with a similar intensity for the reduced enzyme in Fig. 2D. **E**) Quantitative low-molecular-weight mass spectrometry after incubation of CysS_3_Cys with either no enzyme (grey) or reduced PfGrx^MT^ (blue) for 15 min on ice. Data represent three independent experiments using the same enzyme stock (*n* = 3).

**Figure S3**

**Supplementary Figure S3 | Direct rapid reduction of GS_4_G by PfGrx.** **A**) Representative stopped-flow kinetics for the reaction of reduced PfGrx^MT^ (left) or PfGrx^DT^ (right) with variable concentrations of GS_4_G at 25°C and pH 7.4. **B**) Secondary plots for the *k*_obs_ values from single- or double-exponential fits from panel A. The GS_4_G-dependent rate constant for the first phase (left) was determined from the slopes of the secondary plot, whereas the GS_4_G-independent rate constant for the second phase for PfGrx^DT^ (right) was averaged from all data points. Representative traces in panel A were averaged from three technical replicates and *k*_obs_ values in panel B were from a single experiment (*n* = 1×3). **C**) Whole-protein mass spectrometry after incubation of GS_4_G with reduced PfGrx^MT^ or PfGrx^DT^ for 10 s on ice. Calculated masses for glutathionylated PfGrx^MT^ or PfGrx^DT^(S_2_) are 14016 Da or 13725 Da, respectively (following treatment with 0.5 mM HPE-IAM). Additional peaks may arise from oxidation of argininyl to glutamyl residues (−27 Da), methylation (+14 Da), potassium adducts (+38) or alkylation of amines by HPE-IAM (+178 Da). **D**) Quantitative low-molecular-weight mass spectrometry after incubation of GS_4_G with either no enzyme, reduced PfGrxMT or reduced PfGrxDT for 10 s on ice. **E**) Schematic representation of the predicted transition state and assigned rate constant for the reaction between GS_4_G and the reduced enzyme.

**Figure S4**

**Supplementary Figure S4 | Glutathione reductase-dependent formation and reduction of GS_3_H.** **A**) Representative stopped-flow kinetics for the reaction of ScGR with GS_4_G in the presence of variable concentrations of NADPH at 25°C and pH 7.4. **B**) Quantitative low-molecular-weight mass spectrometry after incubation of GS_4_G with either no enzyme, ScGR and 1 eq NADPH (for 10 s on ice) or 2 eq NADPH (for 30 min at 25°C). **C**) Schematic summary of the data interpretation.

**Figure S5**

**Figure S5 | Competition between ScGR and PfGrx^MT^ for the reduction of GS_3_G and GSSH. A**) Schematic summary of the competition assay. GS_3_G can be reduced either by ScGR with NADPH or by PfGrx^MT^ with DTT. Reduction of the intermediate PfGrx^MT^(SSG) by DTT, unlike by GSH, does not lead to the formation of GSSG (and RSSG directly yields the DTT disulfide without complicating data analysis). **B**) Control experiment for the reduction of PfGrx^MT^(SSG) by DTT. Representative stopped-flow kinetics for PfGrx^MT^(SSG) with variable concentrations of DTT at 25°C and pH 7.4. **C**) Secondary plot for the *k*_obs_ values from single exponential fits from panel B. The DTT-dependent rate constant was determined from the slope of the plot. Representative traces in panel B were averaged from three technical replicates and *k*_obs_ values in panel C were generated from three independent biological replicates (*n* = 3×3). The rate constant of 3.4×10^5^ M^−1^s^−1^ is in a similar order of magnitude to that for GSH (Table 1), confirming the suitability of DTT as a reductant for PfGrx^MT^(SSG) in the competition assay. **D**) Representative stopped-flow kinetics for the reduction of GS_3_G by ScGR with NADPH in the presence of DTT and variable concentrations of PfGrx^MT^ at 25°C and pH 7.4. All absorbances were aligned to the measurement without Grx/DTT. A high DTT-concentration of 5 mM was chosen to reflect reducing conditions *in vivo* and to prevent the reduction of PfGrx^MT^(SSG) by much lower concentrations of GSH or H_2_S, formed during GS_3_G reduction. The controls without PfGrx and/or DTT indicate only a minor DTT-mediated ScGR reduction or non-enzymatic reactions between DTT and GS_3_G during the first 5 seconds. Meanwhile, after 5 seconds, reactions between DTT and GSSH are more pronounced. The control with 1 eq Grx without DTT shows that PfGrx^MT^ reduces GSSH about 30 times faster than ScGR under the chosen conditions. In the absence of DTT, the intermediate PfGrx^MT^(SSG) is reduced by GSH yielding GSSG, which is efficiently converted by ScGR. **E**) The NADPH-consumption (Δ[NADPH]) was determined from the amplitudes of panel D and plotted against the [Grx]/[GR] ratio. Phase 1 (left panel) represents the GR-mediated reduction of GS_3_G during the first 5 seconds and phase 2 (right panel) the subsequent reduction of GSSH. The [Grx]/[GR] ratio at half-maximal amplitude (*K*_1/2_) was determined as a parameter of a hyperbolic decay fit. The inverse value (*K*_1/2_)^−1^ suggests that PfGrx^MT^ reduces GS_3_G 1.4 times faster than ScGR and GSSH 18 times faster than ScGR under the chosen conditions. The Δ[NADPH] values in panel E were from two independent experiments (*n* = 2).

**Figure S6**

**Supplementary Figure S6 | Direct rapid reduction of GS_3_H by PfGrx.** **A**) Representative stopped-flow kinetics for the reaction of reduced PfGrx^MT^ (left) or PfGrx^DT^ (right) with variable concentrations of *in situ*-generated GS_3_H at 25°C and pH 7.4. **B–D**) Secondary plots for the *k*_obs_ values from double- or triple-exponential fits from panel A. Actual concentrations may vary, as significant amounts of GSSH are formed besides GS_3_H during the pre-incubation of GS_4_G with GR and NADPH. The GS_n_H-dependent rate constant was determined from the slopes of the secondary plot, whereas the GS_n_H-independent rate constant for the third phase for PfGrx^DT^ and the second phase for PfGrx^MT^ was averaged from all data points. Representative traces in panel A were averaged from three technical replicates and *k*_obs_ values in panel B–D were from a single experiment (*n* = 1×3). **E**) Whole-protein mass spectro-metry after incubation of *in situ*-generated GS_3_H with reduced PfGrx^MT^ or PfGrx^DT^ for 10 s on ice. Calculated masses for reduced and glutathionylated PfGrx^MT^ or PfGrx^DT^(S_2_) are 13888 Da and 14016 Da or 13725 Da, respectively (following treatment with 0.5 mM HPE-IAM). Additional peaks may arise from oxidation of argininyl to glutamyl residues (−27 Da), methylation (+14 Da), potassium adducts (+38) or alkylation of amines by HPE-IAM (+178 Da). **F**) Quantitative low-molecular-weight mass spectrometry after incubation of *in situ*-generated GS_3_H with either no enzyme, reduced PfGrx^MT^ or reduced PfGrx^DT^ for 10 s on ice. **G**) Schematic representation of the predicted transition state and assigned rate constant for the reaction between GS_3_H and the reduced enzyme.

**Figure S7**

**Supplementary Figure S7 | Control experiments for the reduction of GSSH by PfGrx.** **A**) Representative stopped-flow kinetics for the reaction of reduced PfGrx^MT^ with *in situ*-generated GSSH at 25°C and pH 7.4. Even after the addition of 2 eq NADPH, when all GS_3_G should be consumed, a fluorescence change was still observed, indicating that GSSH reacts directly with Grx. **B**) Rate constant for a second reaction phase of PfGrx^MT^ (left) and PfGrx^DT^ (right). **C**) Whole-protein mass spectrometry after incubation of *in situ*-generated GSSH with reduced PfGrx^DT^ for 10 s on ice. Calculated masses for reduced and glutathionylated PfGrx^DT^ or PfGrx^DT^(S)_2_ are 14081 Da and 14032 Da or 13725 Da, respectively (following treatment with 0.5 mM HPE-IAM). Additional peaks may arise from oxidation of argininyl to glutamyl residues (−27 Da), methylation (+14 Da), potassium adducts (+38) or alkylation of amines by HPE-IAM (+178 Da). **D**) Whole-protein mass spectrometry after incubation of Na_2_S with glutathionylated PfGrx^MT^ for 15 min on ice. The calculated mass of reduced PfGrx^MT^ is 13888 Da (following treatment with 2 mM HPE-IAM). **E**) Brønsted plot of the second-order rate constants (triangles) and normalized, pH-independent second-order rate constants (circles) for the reaction of PfGrx^MT^(SSG) with various thiols of differing p*k*_a_-values determined previously.^[6]^ The nucleophile Bronsted coefficient *β*_nuc_ was obtained from the slope of the linear fit. The herein determined rate constant for H_2_S falls slightly below the linear regression, indicating no specificity of Grx(SSG) for H_2_S as a reducing agent compared to other thiols. **F**) Representative stopped-flow kinetics for the reaction of PfGrx^DT^(S)_2_ with various concentrations of Na_2_S (purple traces) at 25°C and pH 7.4. No fluorescence change was observed. The reduction by GSH served as a positive control (blue trace). Pre-incubation with Na_2_S did not significantly change the kinetics with GSH (orange trace), indicating that Na_2_S does not reduce PfGrx^DT^(S)_2_.

**Figure S8**

**Supplementary Figure S8 | SDS-PAGE analyses of representative purifications of recombinant PfGrx variants.** The indicated proteins were separated on a 15% SDS-PAGE gel under reducing conditions and visualized by staining with Coomassie Brilliant Blue. Lanes: M, marker; BI, before induction; AI, after induction; P and S, pellet and supernatant after cell lysis and centrifugation; FT, Ni-NTA agarose flow through; W, Ni-NTA agarose wash fraction; E, Ni-NTA agarose eluate.

**Supplementary References**

[1]	T. Ida, T. Sawa, H. Ihara, et al., “Reactive cysteine persulfides and S-polythiolation regulate oxidative stress and redox signaling” *Proc Natl Acad Sci U S A* **111**, (2014): 7606-7611, DOI: 10.1073/pnas.1321232111.

[2]	C. F. Djuika, S. Fiedler, M. Schnolzer, C. Sanchez, M. Lanzer, M. Deponte, “Plasmodium falciparum antioxidant protein as a model enzyme for a special class of glutaredoxin/glutathione-dependent peroxiredoxins” *Biochim Biophys Acta* **1830**, (2013): 4073-4090, DOI: 10.1016/j.bbagen.2013.04.020.

[3]	N. Guex, M. C. Peitsch, “SWISS-MODEL and the Swiss-PdbViewer: an environment for comparative protein modeling” *Electrophoresis* **18**, (1997): 2714-2723, DOI: 10.1002/elps.1150181505.

[4]	M. Yogavel, T. Tripathi, A. Gupta, et al., “Atomic resolution crystal structure of glutaredoxin 1 from Plasmodium falciparum and comparison with other glutaredoxins” *Acta Crystallogr D Biol Crystallogr* **70**, (2014): 91-100, DOI: 10.1107/S1399004713025285.

[5]	I. Doumi, L. Lang, B. Vileno, M. Deponte, P. Faller, “Glutathione Protects other Cellular Thiols against Oxidation by Cu(II)-Dp44mT” *Chemistry* **30**, (2024): e202304212, DOI: 10.1002/chem.202304212.

[6]	L. Lang, P. Reinert, C. Diaz, M. Deponte, “The dithiol mechanism of class I glutaredoxins promotes specificity for glutathione as a reducing agent” *Redox Biol* **78**, (2024): 103410, DOI: 10.1016/j.redox.2024.103410.

[7]	R. F. Colman, S. Black, “On the Role of Flavin Adenine Dinucleotide and Thiol Groups in the Catalytic Mechanism of Yeast Glutathione Reductase” *J Biol Chem* **240**, (1965): 1796-1803.

[8]	D. Schilling, U. Barayeu, R. R. Steimbach, D. Talwar, A. K. Miller, T. P. Dick, “Commonly Used Alkylating Agents Limit Persulfide Detection by Converting Protein Persulfides into Thioethers” *Angew Chem Int Ed Engl* **61**, (2022): e202203684, DOI: 10.1002/anie.202203684.

[9]	T. Akaike, T. Ida, F. Y. Wei, et al., “Cysteinyl-tRNA synthetase governs cysteine polysulfidation and mitochondrial bioenergetics” *Nat Commun* **8**, (2017): 1177, DOI: 10.1038/s41467-017-01311-y.

[10]	T. Matsunaga, H. Sano, K. Takita, et al., “Supersulphides provide airway protection in viral and chronic lung diseases” *Nat Commun* **14**, (2023): 4476, DOI: 10.1038/s41467-023-40182-4.
